# Insights into Genomic Evolution and the Potential Genetic Basis of Klebsiella variicola subsp. *variicola* ZH07 Reveal Its Potential for Plant Growth Promotion and Autotoxin Degradation

**DOI:** 10.1128/spectrum.00846-22

**Published:** 2022-11-15

**Authors:** Dandan Wang, Li Sun, Zhiqiu Yin, Shiyu Cui, Weiwei Huang, Zhihong Xie

**Affiliations:** a National Engineering Research Center for Efficient Utilization of Soil and Fertilizer Resources, College of Resources and Environment of Shandong Agricultural University, Taian, China; b Department of Clinical Laboratory, The Fifth Affiliated Hospital of Guangzhou Medical University, Guangzhou, China; University of Minnesota

**Keywords:** autotoxin degradation, comparative genomics, *Klebsiella*, peanut, plant growth-promoting rhizobacteria

## Abstract

The accumulation of autotoxins in soil causes continuous cropping obstacle stress in crops, and the bioremediation of autotoxins by microorganisms is an efficient process. In this study, strain ZH07 was isolated from the peanut rhizosphere and was found to be utilizing multiple autotoxins as its carbon sources. Based on its genomic characteristics and a phylogenetic analysis, ZH07 represents a member of Klebsiella variicola subsp*. variicola*. A comparative genomic analysis exhibited evolutionary dynamics exhibited by mobile genetic elements (MGEs), strain-specific genes, potential horizontal genes, and evolutionary constraints driven by purifying selection, which facilitated its genomic adaptation to rhizosphere soil. Genome mining revealed the potential genomic properties associated with plant growth promotion, such as nitrogen fixation, indole acetic acid synthesis, phosphonate solubilization and assimilation, siderophore production, and secondary metabolite synthesis. Moreover, abundant genes putatively responsible for the biodegradation of aromatic xenobiotics, including benzoic acid, cinnamic acid, vanillic acid, protocatechuic acid, phenylacetic acid, and p-hydroxybenzoic acid were also observed in the ZH07 genome. Compared to autotoxin stress alone, the combination of ZH07 application promoted peanut germination and seedling growth. Our analysis revealed the genetic adaptation of ZH07 to the rhizosphere environment and the potential genetic basis and effectiveness of the isolate to serve as a plant growth stimulator.

**IMPORTANCE** Continuous cropping obstacles reduce the production and quality of agricultural products, and the application of rhizosphere beneficial microbes is an important strategy. Strain ZH07 showed autotoxin-degrading and plant growth-promoting capacities. The objectives of this study were to characterize its genomic evolution and the potential genetic basis of the autotoxin degradation and plant growth promotion. ZH07 represents a member of Klebsiella variicola subsp*. variicola*, based on genomic and phylogenetic analyses. Its genomic components have undergone different degrees of purifying selection, and the disparity in the evolutionary rate may be associated with its niche adaptation. A systematic analysis of the ZH07 genome identified the potential genetic basis that contributes to plant growth promotion and to aromatic xenobiotic biodegradation. This study demonstrates that plant growth-promoting rhizobacteria (PGPR) play important roles in autotoxin biodegradation and can be used as biofertilizers to enhance the growth of peanuts in response to continuous cropping obstacle stress.

## INTRODUCTION

Continuous cropping obstacles are caused by planting patterns in which the same or a similar crop is cultivated in the same soil, year after year (1). It reduces the production and quality of agricultural products, seriously restricting the development of modern comprehensive and sustainable agriculture. Importantly, autotoxicity results from the accumulation of autotoxins, which can be released into the environment through root secretion and plant residue decomposition (2–4). The accumulation of autotoxins in soil is harmful to conspecific plants (5–9). Autotoxins can inhibit germination, resulting in oxidative stress in seedling roots, the disruption of membrane permeability, damage to DNA and proteins, the alteration of enzyme activities, and the alteration of mineral uptake (10–14). In addition to the direct impact on plants, the accumulation of autotoxins in the rhizosphere soil can also indirectly promote root disease by changing the microbial community structure, resulting in continuous cropping obstacles (15–17).

Some methods have been used to alleviate autotoxicity, including the selection of resistant varieties, crop rotation and the provision of a proper fallow period for the decomposition of autotoxins (18–21). Notably, the application of environment-friendly, beneficial microbes with highly efficient autotoxin degradation capabilities is increasingly being applied for the suppression of continuous cropping obstacles (22, 23). Among them, plant growth-promoting rhizobacteria (PGPR), as strong root colonizers, represent beneficial functions, including autotoxin degradation, plant growth promotion, and soilborne disease suppression (24–26). A large number of autotoxin-degrading PGPRs have been isolated, including *Bacillus*, *Azotobacter*, Pseudomonas, Staphylococcus, Acinetobacter, and *Exiguobacterium*, among others. Some of them are capable of degrading multiple types of autotoxins in combination with a high degradation efficiency (16, 21, 27, 28). A plant usually produces more than one kind of autotoxin. Hence, the strains with the ability to degrade multiple autotoxins will have a greater practical value. The application of autotoxin-degrading PGPR could be an ecofriendly bioremediation method by which to increase the productivity of plants. For example, Acinetobacter calcoaceticus CSY-P13 and Streptomyces canus GLY-P2 have been reported to rapidly degrade ferulic acids and p-hydroxybenzoic acids and reduce their autotoxicity to cucumbers (16, 28). Moreover, Pseudomonas putida and Pseudomonas hunanensis strains show high efficiency in the degradation of ferulic acid, p-hydroxybenzoic acid, and syringic acid, and they also reduce the inhibition of lily, watermelon, poplar, and strawberry seedling growth (21).

Peanut, as an important oilseed crop worldwide, is sensitive to continuous cropping obstacles. Benzoic acid, p-hydroxybenzoic acid, p-coumaric acid, and several other phenolic acids are the major autotoxins in peanut root exudation (15, 29). In this study, PGPR strain ZH07 isolated from the peanut rhizosphere displayed highly efficient abilities to degrade multiple autotoxins. Whole-genome sequencing (WGS) has offered a tremendous advantage in determining the evolutionary relationship, genetic diversity, and biotechnological properties (30–32). Hence, the genome of ZH07 was sequenced for a comparative genomic analysis. The phylogenetic relationships and evolutionary dynamics were explored to elucidate the evolution of this strain. The key genetic characteristics (e.g., nitrogen fixation, indole acetic acid synthesis, phosphate solubilization and assimilation, siderophore synthesis and transport, secondary metabolite biosynthesis gene clusters, and potential autotoxin degradation pathways) were investigated to reveal the underlying genetic basis of plant growth promotion and autotoxin degradation in the ZH07 genome. The effects of inoculation of ZH07 on peanut seed germination and seedling growth was also evaluated. Our results will provide a new choice for the biological removal of autotoxins and for the alleviation of peanut continuous cropping obstacles.

## RESULTS

### Isolation, identification, and characterization of ZH07.

Using benzoic acid as the sole carbon source, 31 strains were isolated from the peanut rhizosphere as potential autotoxin-degrading strains. The strain ZH07 showed outstanding abilities to degrade autotoxins and promote plant growth. Therefore, it was selected for further studies. Growth of ZH07 occurred at 20 to 50°C (optimum: 30°C) with 0 to 10% NaCl (optimum: 1%) and a pH of 5.0 to 9.0 (optimum: pH 7.0) (Table 1; Fig. S1).

**TABLE 1:** Characterization of Klebsiella ZH07

Characteristic	Klebsiella variicola subsp. *variicola* ZH07
Source	Rhizosphere soil of peanut
Growth condition^*a*^	
Temp range	20 to 50°C (30°C)
NaCl range	0 to 10% (1%)
pH range	5 to 9 (7)
IAA production	8.33 + 0.15 (mg/L)
Siderophore production^*b*^	+
Phosphate solubilization^*b*^	+
Ammonia production^*b*^	+
Potassium solubilization^*b*^	+
Antibiotic susceptibility test^*c*^	Em^+^, Amp^+^, Zeo^+^, Hyg^+^, Gm^−^, Sm^−^, Cm^−^, Kan^−^, Sh^−^, Tc^−^

a Values in parentheses indicate the optimal growth condition.

b +, strain has the related capacity; −, strain does not have the related capacity.

c +, resistant; −, sensitive; Em, erythromycin, 10 ppm; Amp, ampicillin, 100 ppm; Zeo, Zeocin, 20 ppm; Hyg, hygromycin, 30 ppm; Gm, gentamycin, 50 ppm; Sm, streptomycin, 50 ppm; Cm, chloramphenicol, 10 ppm; Kan, kanamycin, 30 ppm; Sh, spectinomycin, 100 ppm; Tc, tetracycline, 15 ppm.

The plant growth-promoting abilities of ZH07 were evaluated, including nitrogen fixation, phosphorus solubilization, potassium dissolution, and siderophore and indole acetic acid (IAA) production (Table 1). In the qualitative assay, ZH07 could grow well in nitrogen-free Ashby medium (Fig. S2A), form a solubilization halo on an inorganic phosphorus medium (Fig. S2B), an egg yolk medium (Fig. S2C), and an Alexandrov medium (Fig. S2D). It also formed orange halo zones, indicating siderophore production (Fig. S2E). Moreover, ZH07 produced 8.33 ± 0.15 mg·L^−1^ of IAA after 4 days of incubation with 200 μg·L^−1^ of l-tryptophan at 30°C (Table 1). In our results, ZH07 showed the capacity of nitrogen fixation, phosphorus solubilization, potassium dissolution, siderophore production, and IAA synthesis, suggesting that ZH07 has the potential to promote plant growth.

### Autotoxin-degrading properties of ZH07.

**FIG 1:**
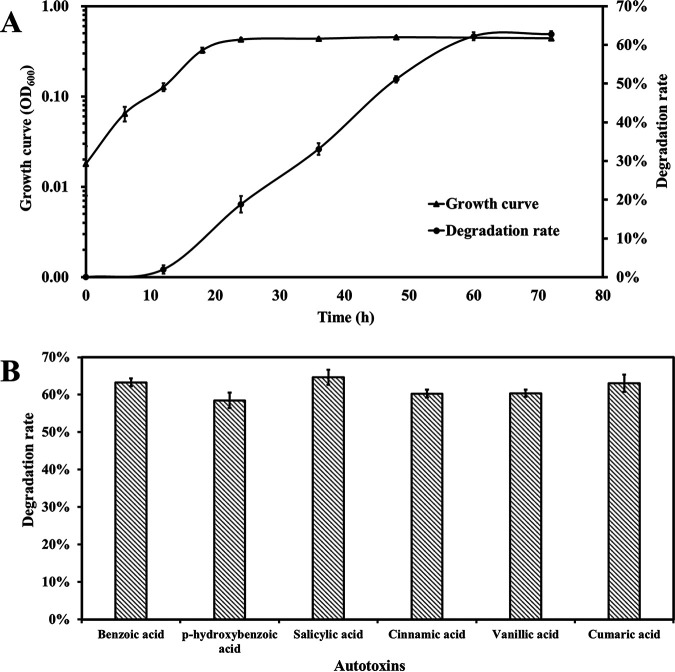
(A) Cell growth in semilogarithmic coordinates and the benzoic acid degradation rate of strain ZH07 in MSM liquid medium. (B) The degradation rates of six phenolic acids by ZH07.

To evaluate its potential for alleviating continuous cropping obstacle stress, the degradation of a range of autotoxins by ZH07 was assessed. As shown in Fig. 1A and in Fig. S3, ZH07 has the ability to grow in MSM medium with benzoic acid as its sole carbon source. It could utilize more than 60% benzoic acid within ~21 h of incubation, and it exhibited maximum growth within 59 h of incubation (Fig. 1A). Moreover, ZH07 was also able to utilize p-hydroxybenzoic acid, salicylic acid, cinnamic acid, vanillic acid, and cumaric acid with more than 50% utilization (Fig. 1B). Hence, our results revealed that ZH07 could efficiently degrade multiple autotoxins, indicating the mitigation effects of ZH07 on autotoxin stress in peanuts.

### Genomic characteristics of ZH07.

**FIG 2:**
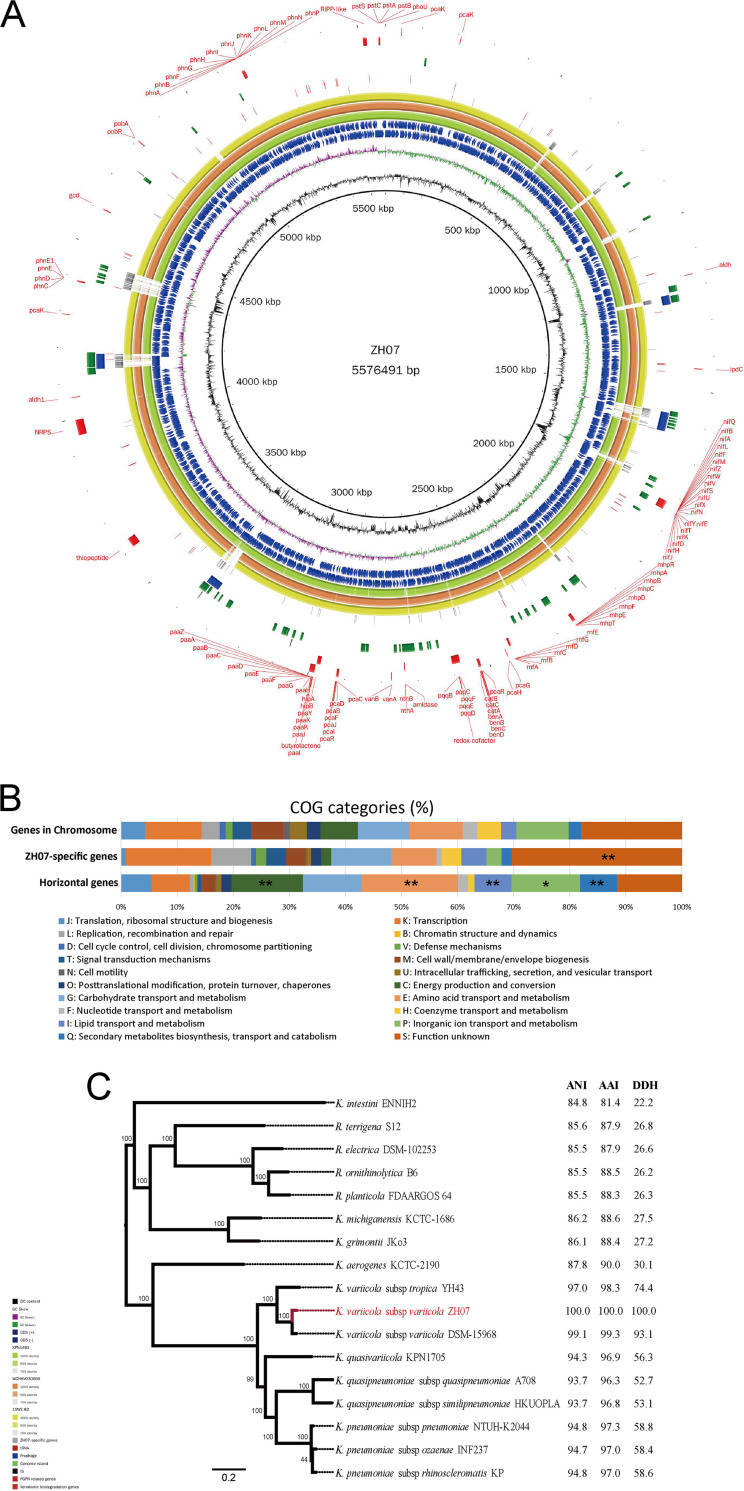
Genome features of the ZH07 chromosome. (A) Circular representation of the ZH07 chromosome. Rings represent the following features labeled from inside to outside: ring 1, GC content; ring 2, GC-skew, where green and purple correspond to above-average and below-average GC-skew, respectively; rings 3 and 4, blue arrows correspond to plus-strand CDS and minus-strand CDS; rings 5 to 7, circular comparison of KPN1481, WCHKV030666, and 15WZ-82, respectively; rings 8 to 14, blocks correspond to ZH07-specific genes, tRNA, prophages, GIs, ISs, secondary metabolite-related biosynthesis gene clusters, and xenobiotic biodegradation genes, respectively. (B) Distribution of COG categories for chromosome genes, ZH07-specific genes, and potential horizontal genes, respectively. *, Fisher’s exact test *P* value < 0.05; **, Fisher’s exact test *P* value < 0.01. (C) Phylogenetic tree based on SNPs across 2,529 single-copy core gene families shared by ZH07 and on 16 complete genomes of other Klebsiella species that was constructed via the maximum likelihood method with 100 replicates.

The above biological properties indicated that the genomic evolution and underlying genetic basis of ZH07 deserve further systematic study. Therefore, the complete genome sequence of ZH07 with one plasmid pZH07 was sequenced. The genome was estimated to have 100% completeness with 1% contamination. The ZH07 chromosome was comprised of 5,576,491 bp with a guanine-cytosine (GC) content of 57.4% and 5,404 rapid antimicrobial susceptibility testing (RAST)-predicted coding sequences (CDSs) (Fig. 2A). Based on the Clusters of Orthologous Genes (COG) functional assignment, a total of 4,886 (90.4%) CDSs were classified into 20 COG categories. “K: transcription” (541 CDSs), “G: Carbohydrate transport and metabolism” (485 CDSs), “E: Amino acid transport and metabolism” (515 CDSs), and “P: Inorganic ion transport and metabolism” (498 CDSs) were the most enriched functional categories in the chromosome (Fig. 2B). About 27.4% of the CDSs were poorly characterized (“S: Functional unknown”: 962 CDSs; “no homologs identified”: 518 CDSs).

The plasmid pZH07 was comprised of 174,413 bp, which made up 3.0% of the ZH07 genome. It harbored 230 RAST-predicted CDSs and presented with a 50.8% GC content. A Nucleotide Basic Local Alignment Search Tool (BLASTN) search of the National Center for Biotechnology Information (NCBI) nr database, using the full plasmid sequence, showed that pZH07 exhibits a backbone similar to those of some described plasmids (e.g., pKLVA-1, pLMG 23571, p15WZ-82_res, pKP91, and pKp5-1) in other *K. variicola* strains (Fig. S4A). The plasmid pZH07 carries a variety of functional genes, including genes related to “K: transcription” (22 CDSs), “L: Replication, recombination and repair” (28 CDSs), and “S: Function unknown” (44 CDSs) (Fig. S4B). Furthermore, one conjugal transfer region (*tra* operon) and one CRISPR locus with 13 different spacer sequences and no *cas* genes was found in pZH07 (Fig. S4A).

In addition to plasmids, several other types of mobile genetic elements (MGEs) were identified in the ZH07 genome, including genomic islands (GIs), prophages, and insertion sequences (ISs) (Fig. 2A; Table S1). A total of 87 tRNA codons were scattered throughout the chromosome. 54 GIs were identified, covering a 541.5 kb (9.7%) region of the chromosome. The ZH07 chromosome harbored three intact prophages and one incomplete prophage that covered a 175.1 kb (3.1%) region of the chromosome. A total of 19 complete IS elements were identified, including 7 on the chromosome and 12 on the plasmid. The presence of diverse MGEs indicated the genetic plasticity of ZH07. We also identified 380 potential horizontal genes (Table S2) in the ZH07 chromosome that were significantly enriched in “C: Energy production and conversion”, “E: Amino acid transport and metabolism”, “I: Lipid transport and metabolism”, “Q: Secondary metabolites biosynthesis, transport and catabolism” (Fisher’s exact test *P* value < 0.01), and “P: Inorganic ion transport and metabolism” (Fisher’s exact test *P* value < 0.05).

### Phylogenetic analysis revealed the evolutionary position of ZH07.

The complete 16S rRNA sequence (1,544 bp) of ZH07 was subjected to similarity-based searches against the taxonomically united 16S rRNA database in EzBioCloud (33). The result (Table S3) revealed up to 99.9% identity with Klebsiella variicola subsp. *tropica* SB5531^T^ (accession number: MK040621.1), as well as high identity matches (>98.0%) with other Klebsiella species (Fig. S4C), indicating that strain ZH07 belongs to the Klebsiella genus. To further explore the taxonomic position of ZH07, a phylogenetic tree was created, based on the 2,529 single-copy core gene families shared by ZH07 and 16 complete genomes of other Klebsiella species (Table S3). The core genome tree identified ZH07 as a member of *K. variicola* subsp. *variicola* (Fig. 2C). The identification is further supported by average nucleotide identity (ANI), average amino acid identity (AAI), and *in silico* DNA-DNA hybridization analyses. The highest ANI (99.1%), AAI (99.3%), and *in silico* DDH (93.1) values were shared between ZH07 and *K. variicola* subsp. *variicola* DSM-15968^T^ (Fig. 2C). Thus, our results indicated that ZH07 is a strain of *K. variicola* subsp. *variicola*.

**FIG 3:**
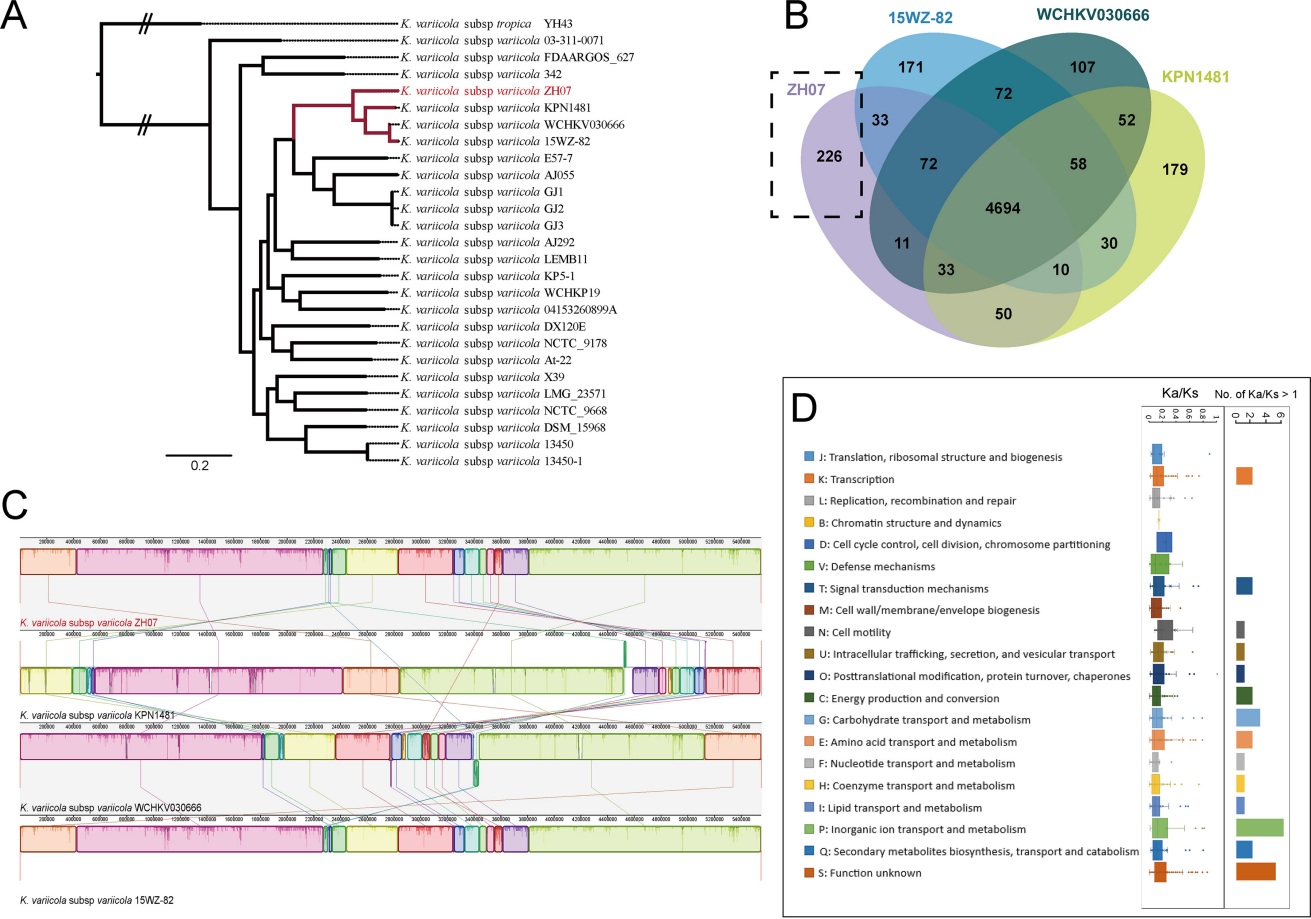
Comparative genomic analysis of ZH07 and closely related strains. (A) Phylogenetic tree based on SNPs across 4,114 single-copy core gene families shared by 27 genomes of *K. variicola* that was constructed via the maximum likelihood method with 100 replicates. (B) Venn diagram displaying overlaps and differences in orthologous gene families in ZH07 and closely related strains (KPN1481, WCHKV030666, and 15WZ-82). (C) Chromosome alignment of ZH07 and closely related strains. Synteny blocks are shown as identically colored regions and are linked across the sequences. (D) Distribution of the *Ka*/*K_s_* rates of the orthologous mutational gene pairs from ZH07 and KPN1481 in COG functional categories.

To further evaluate the evolutionary dynamics of ZH07, 25 publicly complete genomes of *K. variicola* subsp. *variicola* and one complete genome of *K. variicola* subsp. *tropica* as an outgroup were collected for subsequent analyses. A high-resolution phylogeny, based on 4,114 single-copy core gene families that shared all 27 genomes (Table S3), was generated. As shown in Fig. 3A, ZH07, together with KPN1481, WCHKV030666, and 15WZ-82, formed a monophyletic clade and were deeply nested within the tree, indicating their close evolutionary relationship. Unlike ZH07, which was isolated from the rhizosphere soil, KPN1481, WCHKV030666, and 15WZ-82 were isolated from Homo sapiens. Considering the difference in niches, a comparative genome analysis of these strains might provide insights into the specific adaptation and genomic evolution of ZH07.

### Comparative genomic analysis of ZH07 with closely related species revealed its evolutionary dynamics.

We performed a comparative genome analysis of ZH07 with these closely related strains. A total of 226 ZH07-specific gene families (226 genes) were identified (Fig. 3B). As shown in Fig. 2A, additional features in these ZH07-specific genes, such as adjacent tRNAs and insertion sequences, as well as remnants of genomic islands and prophages, indicated the occurrence of horizontal gene transfers (HGTs). It can be assumed that HGTs were evolutionary forces that contributed to the genome-specific evolution of ZH07. The COG classification showed that the genes unique to ZH07 were significantly enriched in “S: Function unknown” (Fisher’s exact test *P* value < 0.01) (Fig. 2B). These unknown functional genes in the ZH07-specific genetic repertoires might play a role in the niche adaptation of ZH07 and in their biological functions, but this requires further research. To further elucidate the evolutionary dynamics of ZH07 with these closely related strains, chromosome rearrangement and synteny analysis were performed. Most regions of the ZH07 chromosome appeared as synteny blocks (*n* = 16), spanning 5,549,018 bp (99.5%) (Fig. 3C). We did not observe significant rearrangements and inversions occurring in the genomic evolution of these strains.

To explore the selective pressure of genetic function, the evolutionary signatures of 892 orthologous mutational gene pairs between ZH07 and KPN1481 were characterized by their nonsynonymous (*Ka*) and synonymous (*K_s_*) substitution rates (*Ka*/*K_s_*). Overall, the average *Ka*/*K_s_* rate of these gene pairs was 0.213 ± 0.374. The *Ka*/*K_s_* rates of most of the gene pairs (*n* = 866; 97.1%) were less than 1. Our analysis exhibited a predominant action of purifying selection. We further investigated the degree of purifying selection of each functional category. The genes related to “M: Cell wall/membrane/envelope biogenesis” (average *Ka*/*K_s_ *= 0.117 ± 0.106), “L: Replication, recombination and repair” (average *Ka*/*K_s_ *= 0.142 ± 0.149), and “F: Nucleotide transport and metabolism” (average *Ka*/*K_s_ *= 0.148 ± 0.232) exhibited stronger evolutionary constraints of purifying selection (Fig. 3D). In contrast, the genes involved in “N: Cell motility” (average *Ka*/*K_s_ *= 0.406 ± 0.709), “T: Signal transduction mechanisms” (average *Ka*/*K_s_ *= 0.315 ± 0.698), and “P: Inorganic ion transport and metabolism” (average *Ka*/*K_s_ *= 0.259 ± 0.441) underwent weaker evolutionary constraints of purifying selection (Fig. 3D). A total of 26 gene pairs were identified under positive selection (*Ka*/*K_s_* > 1) (Table S4). Most of the positively selected genes were distributed in functional categories that were undergoing weaker purifying selection, such as “P: Inorganic ion transport and metabolism” (*n* = 6), “S: Function unknown” (*n* = 5), “T: Signal transduction mechanisms” (*n* = 2), and “N: Cell motility” (*n* = 1) (Fig. 3D). Considering the differences in the niches of the two strains (ZH07: rhizosphere soil; KPN1481: Homo sapiens), it can be inferred that the weaker functional constraints of “N: Cell motility” and “T: Signal transduction mechanisms” might promote adaptation during habitat conversion.

### Genomic properties associated with plant growth-promotion.

**FIG 4:**
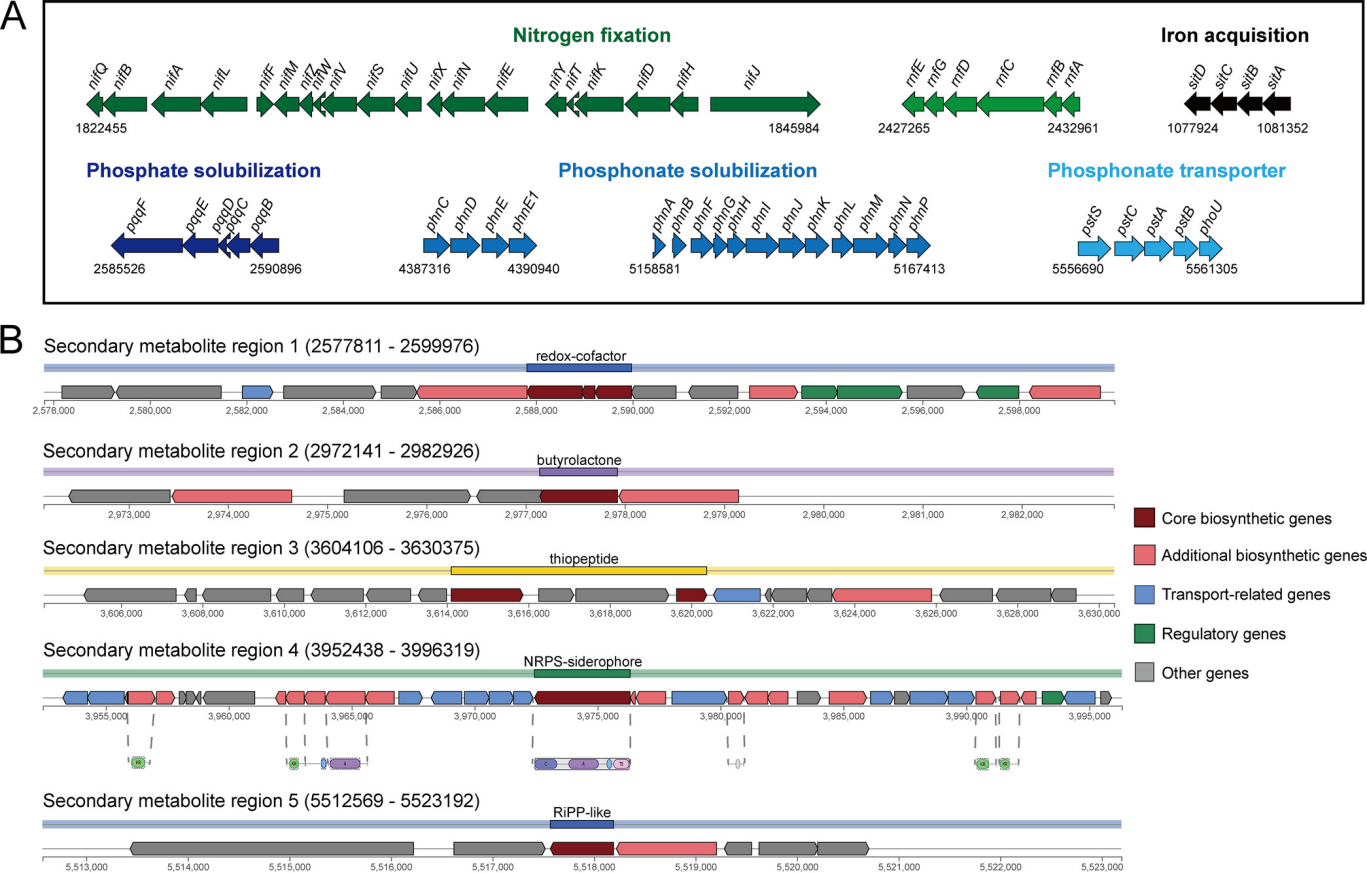
Genomic characteristics of ZH07 associated with plant growth promotion. (A) Genetic organization of the related gene clusters in the ZH07 chromosome. (B) Genetic organization of the gene clusters for secondary metabolism in the ZH07 chromosome.

Regarding nitrogen fixation, the ZH07 genome harbored a *nif* operon, consisting of 20 genes (*nifQBALFMZWVSUXNEYTKDHJ*), arranged within a 23.5 kb region in the chromosome (Table S5; Fig. 4A). Most of them were shown to be required for the synthesis and activity of nitrogenase, the key enzyme in nitrogen fixation (34). The *rnf* operon (*rnfABCDGE*), encoding a membrane-bound protein complex related to the transport of electrons to nitrogenase, was also found in the ZH07 chromosome (Table S5; Fig. 4A) (35). Strain ZH07 has the ability to produce IAA (Table 1). Bacterial IAA synthesis is mainly from tryptophan via four main alternative pathways, including the indole-3-pyruvic acid (IPyA), indole-3-acetamide (IAM), indole-3-acetonitrile (IAN), and tryptamine (TAM) pathways (36). In ZH07, we identified *ipdC* (encoding indolepyruvate decarboxylase, a key enzyme in the IPyA pathway), *aldh* (encoding aldehyde dehydrogenase, converting Indole-3-acetaldehyd to IAA), *nthAB* (encoding nitrile hydratase, converting IAN to IAM), and *amiE* (encoding amidase, converting IAM to IAA) (Table S5).

Relating to phosphate solubilization and assimilation, rhizobacteria could produce organic acids (mainly gluconic acid) to solubilize insoluble or poorly soluble mineral phosphates (37). Gluconic acid dehydrogenase encoded by *gad* and cofactor pyrrolo-quinolone quinine (PQQ) encoded by the *pqq* operon play a key role in the synthesis of gluconic acid (38, 39). In ZH07, the *pqq* operon (*pqqBCDEF*) and the *gcd* gene were identified. We also found two *phn*-related operons (*phnCDEE* and *phnABFGHIJKLMNP*) in the chromosome, which were reported to be responsible for the uptake and degradation of phosphonate (organophosphorus molecules) (40). Furthermore, the *pst* operon (*pstSCAB*), encoding a high-affinity free-phosphate transport system, was present in ZH07 (41).

Our genomic analysis also identified five putative biosynthesis gene clusters (BGC 1 to 5) associated with the synthesis of secondary metabolites (Fig. 4B; Fig. S5; Table S6). BGC-1 was predicted to synthesize the redox-cofactor and exhibited similarity to the biosynthesis regions of lankacidin C, which exhibit antimicrobial activities (42). BGC-2 might be responsible for butyrolactone biosynthesis, as butyrolactone derivatives were reported to show pronounced antimicrobial activities (43). BGC-3 might be associated with the synthesis of thiopeptides (a class of natural product antibiotics) (44). BGC-5 was predicted to synthesize RiPP-like peptides (BGC-5). RiPPs had various biological functions, such as antibacterial, antifungal, and cytotoxic activities (45). Thus, it can be inferred that these BGCs might play a possible role in ZH07 in plant disease biocontrol. BGC-4 harbored several genes encoding the synthesis and transport of siderophores, such as *entABCDEHFS* for the synthesis and transport of enterobactin and *fepABCDG* encoding siderophores receptors (46, 47). Indeed, our results indicated that strain ZH07 could synthesize siderophores. Furthermore, ZH07 also carried iron acquisition genes, such as *sitABCD* (Fig. 4A) (48).

### Genomic insight into the biodegradation of aromatic xenobiotics in ZH07.

**FIG 5:**
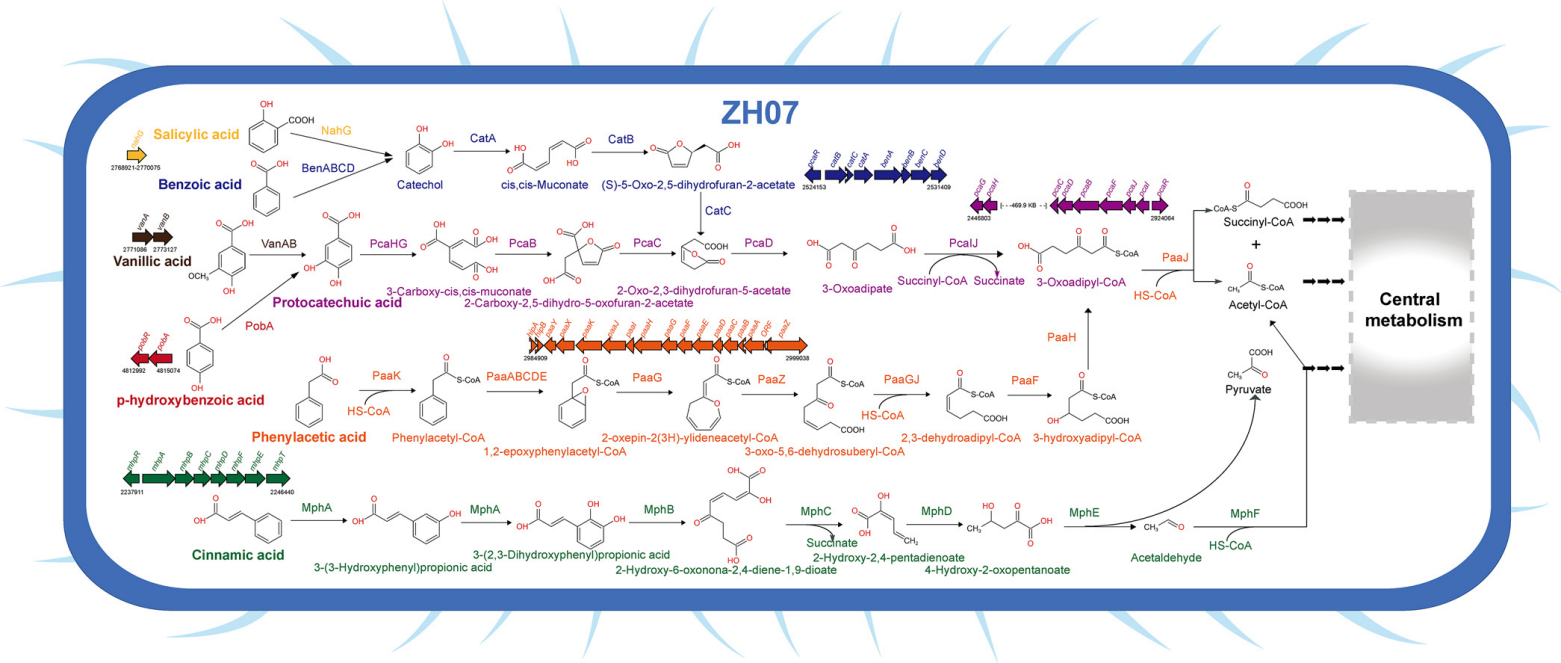
Genomic characteristics of ZH07 related to aromatic xenobiotic biodegradation. The cell cartoon was constructed from the genome annotation of ZH07. The features putatively involved in the aromatic xenobiotic biodegradation are shown. The genetic organization of the related gene clusters in the ZH07 chromosome is shown.

The ZH07 genome was further analyzed with respect to the genetic elements potentially involved in the aromatic xenobiotic biodegradation. In total, several genetic loci determined in the chromosome played a putative role in the biodegradation of several aromatic compounds, including protocatechuic acid, *p*-hydroxybenzoic acid, vanillic acid, benzoic acid, salicylic acid, phenylacetic acid, and cinnamic acid. A variety of aromatic compounds can be converted to central intermediates, including protocatechuate or catechol. Herein, the ZH07 genome possessed the complete protocatechuate branch of the β-ketoadipate pathway, which consists of the *pca*I (*pcaHG*) and *pca*II (*pcaCDBFJIR*) operons, which encode enzymes for the catabolism of protocatechuic acid to succinate and 3-oxoadipyl-CoA (Fig. 5) (49). Environmental bacteria could use the protocatechuate branch of the β-ketoadipate pathway for the degradation of various aromatic pollutants. In ZH07, we identified the *van* (*vanAB*) and *pob* (*pobRA*) operons, which mediated the degradation of vanillic acid and p-hydroxybenzoic acid, respectively (Table S7). The two-component vanillate O-demethylase encoded by *vanAB* transforms vanillic acid to protocatechuic acid (50). The *pobA* gene encoded a hydroxylase that converts *p*-hydroxybenzoic acid to protocatechuic acid (51). The adjacent *pobR* gene encoded an AraC family transcriptional regulator PobR as a transcriptional activator of *pobA* (52). Furthermore, three open reading frames (ORFs) (*pcaK*) encoding a major facilitator superfamily (MFS) transporter (AAHS family) were found in the ZH07 chromosome, and these could play a potential role in the transport of *p*-hydroxybenzoic acid (53).

The catechol branch of the β-ketoadipate pathway was also identified in ZH07, including the *benABCD* and *catBCA* operons. In the catechol branch, benzoic acid is converted into 1,2-dihydro-1,2-dihydroxybenzoic acid by benzoate-1,2-dioxygenase (*benABC*) and is then transformed into catechol by *cis*-diol dehydrogenase (*benD*) (Fig. 5; Table S7) (54). Subsequently, the metabolites of each process in the catechol branch undergo a series of transformations by functional enzymes encoded by *catA*, *catB*, and *catC* and are finally converted into 2-oxo-2,3-dihydrofuran-5-acetate that can be further catabolized by enzymes from the *pca* operon (Fig. 5) (55). Furthermore, the predicted protein encoded by the *nahG* gene (ORF2729) exhibited high identity (98.7%) with the annotated salicylate hydroxylase (AHM85232) from K. pneumoniae 30660. You et al. have reported that salicylate hydroxylase catalyzed the catabolism of salicylic acid through its conversion to catechol (56).

In addition to the β-ketoadipate pathway, other aromatic compound degradation-related elements were also identified in ZH07. Phenylacetate is a major intermediate in the degradation of the diverse aromatic compounds of environmental bacteria. Herein, the *paa* operon (*paaYXKJIHGFEDCBAZ*), comprising the phenylacetic acid catabolic pathway, was arranged within a 13.5 kb region in the ZH07 chromosome (Table S7). These genes encode functional enzymes for the transformation of phenylacetic acid to succinyl-CoA and acetyl-CoA, which are subsequently fed to the central metabolism (Fig. 5) (57). Regarding cinnamic acid degradation, we also identified the *mhp* operon (*mhpRABCDEFET*) encoding functional enzymes that mediate the degradation of 3-(3-hydroxyphenyl)propionic acid to pyruvate and acetyl-CoA (Fig. 5) (58). Based on the KEGG annotation (M00545 *trans*-cinnamate degradation), *trans*-cinnamic acid could be converted to 3-(3-hydroxyphenyl)propionic acid (Reaction: R06781) by hydroxylase MphA (K05712). However, recent research indicated that MphA in Escherichia coli K-12 showed no activity in response to *trans*-cinnamic acid and *cis*-cinnamic acid (59). Thus, the cinnamic acid catabolic pathway in ZH07 deserves further study in future work. Overall, the existence of these genes, carried by the ZH07 genome, provides a potential genomic basis for xenobiotic degradation, whereas the detailed elucidation of how they function requires further transcription and mutation studies.

### Effects of ZH07 application on peanut seed germination and seedling growth.

**FIG 6:**
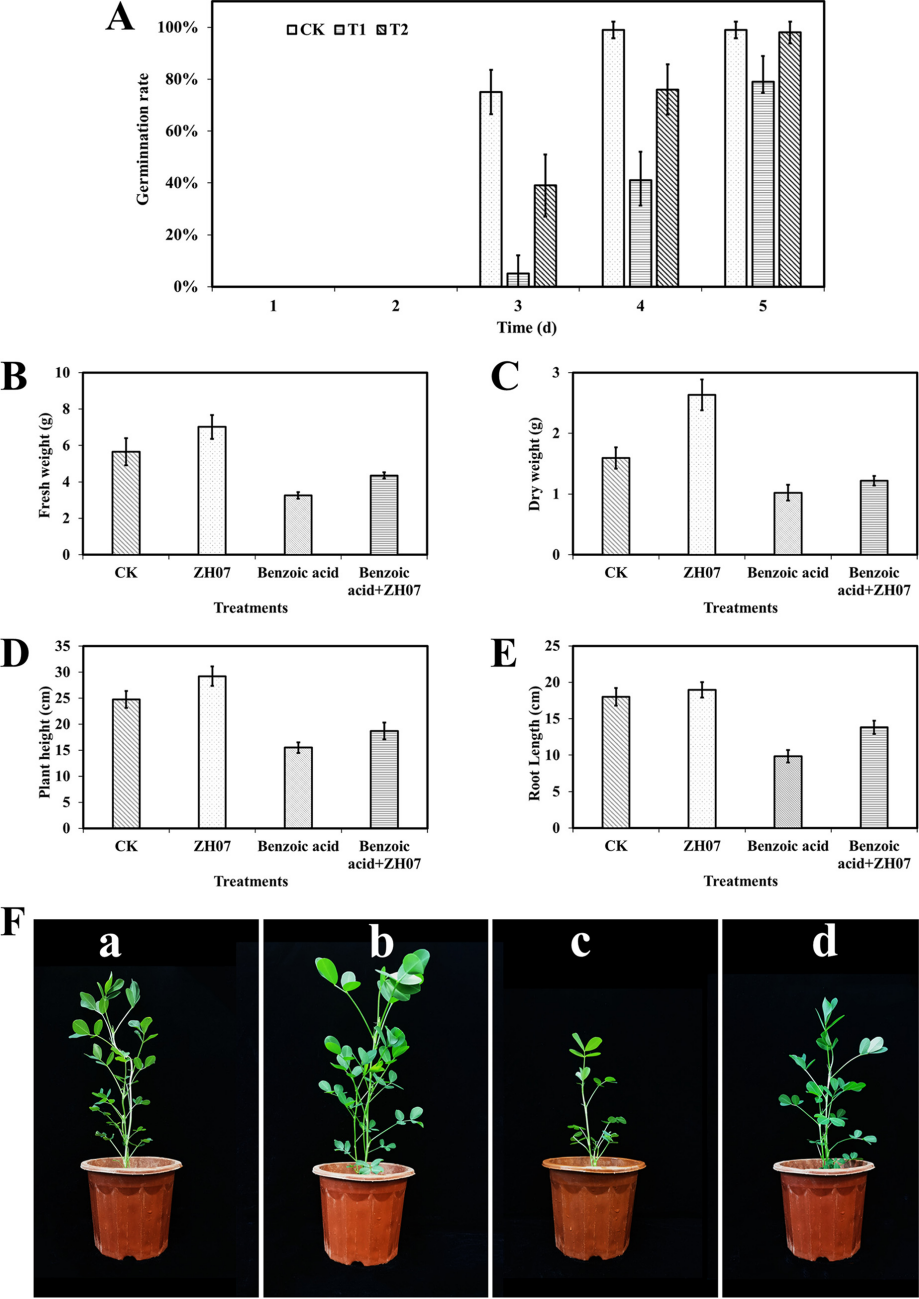
Bioassay on peanut of benzoic acid and the detoxification effect by strain ZH07. (A) Effects of ZH07 and benzoic acid application on peanut seed germination. CK, the surface-sterilized seeds were treated with distilled water; T1, the surface-sterilized seeds were treated with 200 mg·L^−1^ of benzoic acid; T2, the surface-sterilized seeds were treated with 200 mg·L^−1^ of benzoic acid and ZH07 suspensions (10^8^ cells·mL^−1^). Effects of ZH07 and benzoic acid on peanut seeding on fresh weight (B), dry weight (C), plant height (D), and root length (E). (F) Pot experiment. (F, panel a) CK, plants were just watered with Murashige Skoog (MS) medium. (F, panel b) ZH07, watered with 20 mL of ZH07 suspensions (10^8^ cells·mL^−1^). (F, panel c) Benzoic acid, watered with 20 mL of benzoic acid solution (4 g·L^−1^) to make the benzoic acid concentration reach 200 μg·g^−1^ soil. (F, panel d) Benzoic acid+ZH07 treatment group, the seedlings were watered with autoclaved MS liquid medium.

The toxicity of benzoic acid and the detoxification effect of ZH07 were evaluated using peanut seeds (Fig. 6A). On the third day, when peanut seeds were treated with distilled water (CK), the germination rate was 75% at 3 d, but the germination rate of seeds exposed to benzoic acid was reduced by 72% (T1) (*t* test, *P* < 0.01); however, when the peanut seeds were exposed to benzoic acid inoculated with strain ZH07 (T2), the germination rate increased by 36% compared with T1 (*t* test, *P* < 0.01).

The growth-promoting capacity of strain ZH07 was further studied in a pot experiment. Compared to a control, the fresh weight (Fig. 6B), dry weight (Fig. 6C), plant height (Fig. 6C), and root length (Fig. 6D) of peanut seedilngs were increased significantly in the ZH07 treatment by 24.3% (*t* test, *P* value < 0.01), 65.4% (*t* test, *P* value < 0.01), 18% (*t* test, *P* value < 0.01), and 5.2%, respectively, but they decreased when exposed to benzoic acid by 42.4% (*t* test, *P* value < 0.01), 35.8% (*t* test, *P* value < 0.01), 37.4% (*t* test, *P* value < 0.01), and 45.4% (*t* test, *P* value < 0.01), respectively. Meanwhile, under benzoic acid conditions, strain ZH07 could also increase the fresh weight, dry weight, plant height, and root length by 33.8% (*t* test, *P* value < 0.01), 19.4%, 20.8% (*t* test, *P* value < 0.01), and 40.7% (*t* test, *P* value < 0.01), respectively. Pot experiments further confirmed that strain ZH07 could promote the growth of peanuts (Fig. 6F) and exhibited a significant detoxification effect.

## DISCUSSION

### Conclusions.

This study provided a comprehensive understanding of a novel PGPR strain, ZH07, that was isolated from peanut rhizosphere soil. This strain could not only degrade benzoic acid but also exhibit efficient p-hydroxybenzoic acid, salicylic acid, cinnamic acid, vanillic acid, and cumaric acid degradation. Moreover, in a plant growth-promoting attributes assay, ZH07 showed the capacities of nitrogen fixation, phosphorus solubilization, potassium dissolution, siderophore production, and IAA synthesis. We report the complete genome and comprehensive comparative analysis of this strain. The phylogenetic analysis of the 16S rRNA and the core genome in combination with the ANI, AAI, and *in silico* DDH values determined the accurate evolutionary position of ZH07, which represents a member of *K. variicola* subsp*. variicola*. A comparative genomic analysis of ZH07 with closely related strains showed the evolutionary dynamics exhibited by the existence of strain-specific genes related to diverse MGEs (e.g., ISs, plasmids, prophages, and GIs) as well as a predominant action of purifying selection. Different degrees of purifying selection occurred in the gene families with different functional categories. Furthermore, abundant genes putatively responsible for plant growth promotion and aromatic xenobiotic biodegradation were determined. Meanwhile, we also demonstrated that the inoculation of ZH07 could promote peanut germination and seedling growth against the toxic effects of benzoic acid. These results provide a comprehensive understanding of the genomic evolution of ZH07 and provide insights into the adaptive evolution of the PGPR strain in rhizosphere habitats. This study provided a theoretical reference for PGPR commercialization as a potential candidate for various bioremediation applications.

## MATERIALS AND METHODS

### Isolation of the strain ZH07.

Rhizosphere soil was collected from an agricultural field at Qingdao County, Shandong Province, China (36°34' and 120°12'E), where peanuts have been continuously planted for 12 years. Mineral salt medium (MSM) (1L: K_2_HPO_4_, 58 g; (NH_4_)_2_SO_4_, 20 g; KH_2_PO_4_, 45 g; CaCl_2_, 0.002 g; MgCl_2_, 0.16 g; FeCl_3_, .00018 g; Na_2_MoO_4_·2H_2_O, .00024 g; MnCl_2_,·2H_2_O, 0.00015 g; pH 7.0) containing benzoic acid as the sole carbon source was used to isolate degrading bacteria. One gram of a rhizosphere soil sample was added to 100 mL MSM medium supplemented with 1 g·L^−1^ benzoic acid and then incubated at 30°C and 180 rpm for 7 days for enrichment. Then, the strain ZH07 was isolated from the enriching medium and stored in the China General Microbiology Culture Collection Center (CGMCC accession number: 21261).

### Autotoxin degradation properties of ZH07.

ZH07 was grown at 30°C in Luria-Bertani (LB) medium (1L: peptone, 10 g; yeast extract, 5 g; NaCl, 5 g; pH 7.0) solidified with 2.5% agar. A single colony from the LB plate was inoculated into liquid MSM and incubated at 30°C and 180 rpm to the logarithmic phase, and the bacterial cells were collected, washed with liquid MSM, and resuspended in liquid MSM to an OD_600_ of 1.0. Then, 1 mL of this culture was inoculated into 100 mL MSM containing 1 g·L^−1^ of benzoic acid and incubated for 72 h at 30°C and 180 rpm. The control group was uninoculated, and then the benzoic acid residue was quickly analyzed using a microplate reader (SpectraMax 190, Molecular Devices Corporation, American) at 227 nm. The degradation rate was calculated by using the light absorptance value compared to the control, and the tests of the other autotoxin degradation abilities of ZH07 (p-hydroxybenzoic acid, salicylic acid, cinnamic acid, vanillic acid, cumaric acid) were tested using previously described methods (60).

### Plant growth-promoting attributes.

Nitrogen fixation activity was tested on N-free Ashby medium (1L: mannitol, 10.0 g; KH_2_PO_4_, 0.2 g; MgSO_4_, 0.2 g; NaCl, 0.2 g; CaSO_4_, 0.1 g; CaCO_3_, 5 g; pH 7.0, solidified with 2.5% agar). ZH07 was grown at 30°C on Ashby medium for 7 days, and then the growth of the colony was observed.

The mineral phosphate solubilization activity was assayed on inorganic phosphorus medium (1L: glucose, 10 g; (NH_4_)_2_SO_4_, 0.5 g; NaCl, 0.3 g; KCl, 0.3 g; FeSO_4_,·7H_2_O 0.003 g; MnSO_4_·4H_2_O, 0.003 g; MgSO_4_·7H_2_O, 0.3 g; Ca(PO_4_)_2_, 5 g; pH 7.0; solidified with 2.5% agar). The organic phosphate solubilization potential was assayed on egg yolk medium (1L: glucose, 10 g; (NH_4_)_2_SO_4_, 0.5 g; NaCl, 0.3 g; KCl, 0.3 g; FeSO_4_·7H_2_O, 0.003 g; MnSO_4_·4H_2_O, 0.003 g; egg yolk lecithin, 0.2 g; CaCO_3_, 5 g; yeast extract, 5 g; pH 7.0; solidified with 2.5% agar). ZH07 was grown at 30°C on inorganic phosphorus medium or egg yolk medium for 7 days. The phosphate solubilization capacity was characterized by a clear halo around the bacterial colonies.

The potassium dissolution activity was assayed on Alexandrov medium (61) (1L: glucose, 5 g; Na_2_HPO_4_, 2 g; MgSO_4_·7H_2_O, 0.5 g; FeCl_3_, 0.005 g; CaCO_3_, 0.1 g; potassium feldspar powder, 1 g; pH 7.0; solidified with 2.5% agar). ZH07 was grown at 30°C on Alexandrov medium for 7 days. The potassium dissolution capacity was characterized by a clear halo around the bacterial colonies.

The siderophore production activity was tested on chrome azurol S assay medium (1L: CAS, 60.5 mg; HDTMA, 72.9 mg; FeCl·6H_2_O 2.645 mg; NaH_2_PO_4_·2H_2_O, 295.25 mg; Na_2_HPO_4_·12H_2_O, 1213.5 mg; NH_4_Cl, 125 mg; KH_2_PO_4_, 37.5 mg; NaCl, 62.5 mg; pH 7.0; solidified with 2.5% agar) (62). ZH07 was grown at 30°C on chrome azurol S assay medium for 7 days. The siderophore production capacity was characterized by orange color-change reactions on the blue agar around the bacterial colonies.

Indole acetic acid (IAA) production was studied according to Salkowski’s method (63). ZH07 were incubated in LB medium containing 200 μg·L^−1^ l-tryptophan at 30°C and 180 rpm for 4 days. The supernatant was collected by centrifugation at 4°C and 8,000 rpm for 10 min. Then, 2 mL of the supernatant was mixed with 2 mL of Salkowski reagent (50 mL, 35% of perchloric acid, 1 mL 0.5 mol·L^−1^ FeCl_3_ solution) and then incubated in darkness for 30 min and quickly analyzed using the spectrophotometer at OD_530_. The levels of IAA were calculated via comparison with the standard curve.

### Seed germination.

Seed germination tests were carried out using peanut seeds cultivar Huayu-20. Peanut seeds were surface sterilized using 5% sodium hypochlorite for 10 min and then washed with sterilized distilled water three times. ZH07 was grown in LB liquid medium at 30°C for an OD_600_ value of 1.0 and then centrifuged at 4°C and 8,000 rpm to obtain cell pellets. The pellets were washed with sterilized distilled water three times and resuspended in the same volume of sterile distilled water. The experiment includes three treatments: CK, the surface-sterilized seeds were treated with distilled water; T1, the surface-sterilized seeds were treated with 200 mg·L^−1^ of benzoic acid; T2, the surface-sterilized seeds were treated with 200 mg·L^−1^ of benzoic acid and ZH07 suspensions (10^8^ cells·mL^−1^). Peanut seeds were placed in a 9 cm diameter petri dish that contained two layers of sterile filter paper. 10 seeds were placed in each dish, and each treatment was replicated five times.

### Pot experiment.

Peanut (Huayu-20) seeds were surface-sterilized and grown in nursery cups and then transplanted to pots filled with 400 g of soil-less growth media (Klasmann-Deilmann Base Substrate, Recipe-No. 422, blended with sterile vermiculite, 1:1) at four true leaves of age. The experiment includes four treatments: CK, plants were watered with Murashige Skoog (MS) medium; ZH07, ZH07 treatment group, watered with 20 mL of ZH07 suspensions (10^8^ cells·mL^−1^); Benzoic acid, benzoic acid treatment group, watered with 20 mL of benzoic acid solution (4 g·L^−1^) so as to make the benzoic acid concentration reach 200 μg·g^−1^ soil; Benzoic acid + ZH07 treatment group, the seedlings were watered with autoclaved MS liquid medium and grown under conditions of 70% humidity with 25 ± 2°C during the day (12 h, 600 μmol·m^−2^·s^−1^) and 22 ± 2°C during the night (12 h) for 15 days.

### Genome sequencing and bioinformatics analysis.

The ZH07 genome was sequenced using a PacBio RS II platform and an Illumina HiSeq 4000 platform at the Beijing Genomics Institute (BGI, Shenzhen, China). Draft genomic contigs were assembled using a Celera Assembler against a high-quality corrected circular consensus sequence subreads set. The estimates for genome completeness and contamination were performed using CheckM (64). The genome sequence was annotated using the Rapid Annotations using Subsystems Technology (RAST) (65). The genome data were deposited in the NCBI GenBank (accession number: GCA_020995405.1). The features of chromosomes and plasmids, as well as the comparisons thereof, were performed using the BLAST Ring Image Generator (BRIG) (66). The IslandViewer 4 was used for the genomic islands analysis (67). The prophage regions were predicted using the PHAge Search Tool - Enhanced Release (PHASTER) (68). The annotations of the insertion sequences were obtained using the ISFinder (69). The Clusters of Orthologous Groups (COG) and Kyoto Encyclopedia of Genes and Genomes (KEGG) databases were used for the general functional annotations (70). The ZH07 chromosome was analyzed for the presence of horizontal genes using HGTector (71). Klebsiella (rank, genus; taxon ID: 570) and *Enterobacteriaceae* (rank, family; taxon ID: 543) were set as the self-group and close-group, respectively. A syntenic analysis was conducted via ProgressiveMauve (72), which enabled the identification of locally colinear blocks (LCBs) using default parameters. For the selection pressure analysis, ParaAT was used to codon-based align the orthologous genes between ZH07 and *K. variicola* subsp. *variicola* KPN1481 (73). The KaKs_Calculator 2.0 was used to compute the nonsynonymous (*Ka*) and synonymous (*K_s_*) substitution rates (*Ka*/*K_s_*) (74). The gene cluster that was related to secondary metabolism was identified and analyzed using antiSMASH (75).

### Phylogenetic analysis.

The 16S rRNA sequence of ZH07 was compared with the taxonomically united 16S rRNA database in EzBioCloud (33) to identify similar species, and the 16S rRNA and genome sequences of these species were collected for subsequent analyses. The collected genomes were reannotated using the RAST (65). The average nucleotide identity, amino acid identity, and *in silico* DNA-DNA hybridization were calculated using JSpecies 1.2.1 (76), CompareM, and the genome-to genome distance calculator 3.0 (GGDC) (77), respectively. For the core genome phylogeny, orthologous groups of protein families were delimited using the OrthoFinder software package (78). The single-copy core gene families were extracted from the OrthoFinder output files. The nucleotide sequences of the single-copy core gene families were extracted according to the protein accession numbers and were then aligned using the MAFFT software package (79). The core genome tree was constructed using the set of single-nucleotide polymorphisms (SNPs) that were present in the single-copy core gene families. The set of SNPs was integrated according to the arrangement of the genes on the ZH07 chromosome. The putative recombinational regions were identified and removed from the set of SNPs using the ClonalFrameML software package (80). The maximum likelihood (ML) tree was constructed using the MEGA X software package (81) with the general time-reversible (GTR) model with 100 bootstrap replicates.

### Statistical analysis.

Data were expressed as means ± standard errors. All analyses were performed using the SPSS v22.0 software package, and the differences were analyzed by using a one-way analysis of variance (ANOVA) and the least significant difference (LSD) test. The statistical significance of differences (*P* < 0.05) was assessed.

### Data availability.

The genome data of ZH07 were deposited in NCBI GenBank (accession number GCA_020995405.1).
