# Supplementary material for: Insights into Genomic Evolution and the Potential Genetic Basis of Klebsiella variicola subsp. variicola ZH07 Reveal Its Potential for Plant Growth Promotion and Autotoxin Degradation
Source: Microbiol Spectr. 2022 Nov 15;10(6):e00846-22. doi: 10.1128/spectrum.00846-22 (PMC9769570; doi:10.1128/spectrum.00846-22)
Supplement: Supplemental file 1 — Fig. S1 to S5. Download spectrum.00846-22-s0001.pdf, PDF file, 1.0 MB [file spectrum.00846-22-s0001.pdf]

SUPPLEMENTAL MATERIAL FIGURE

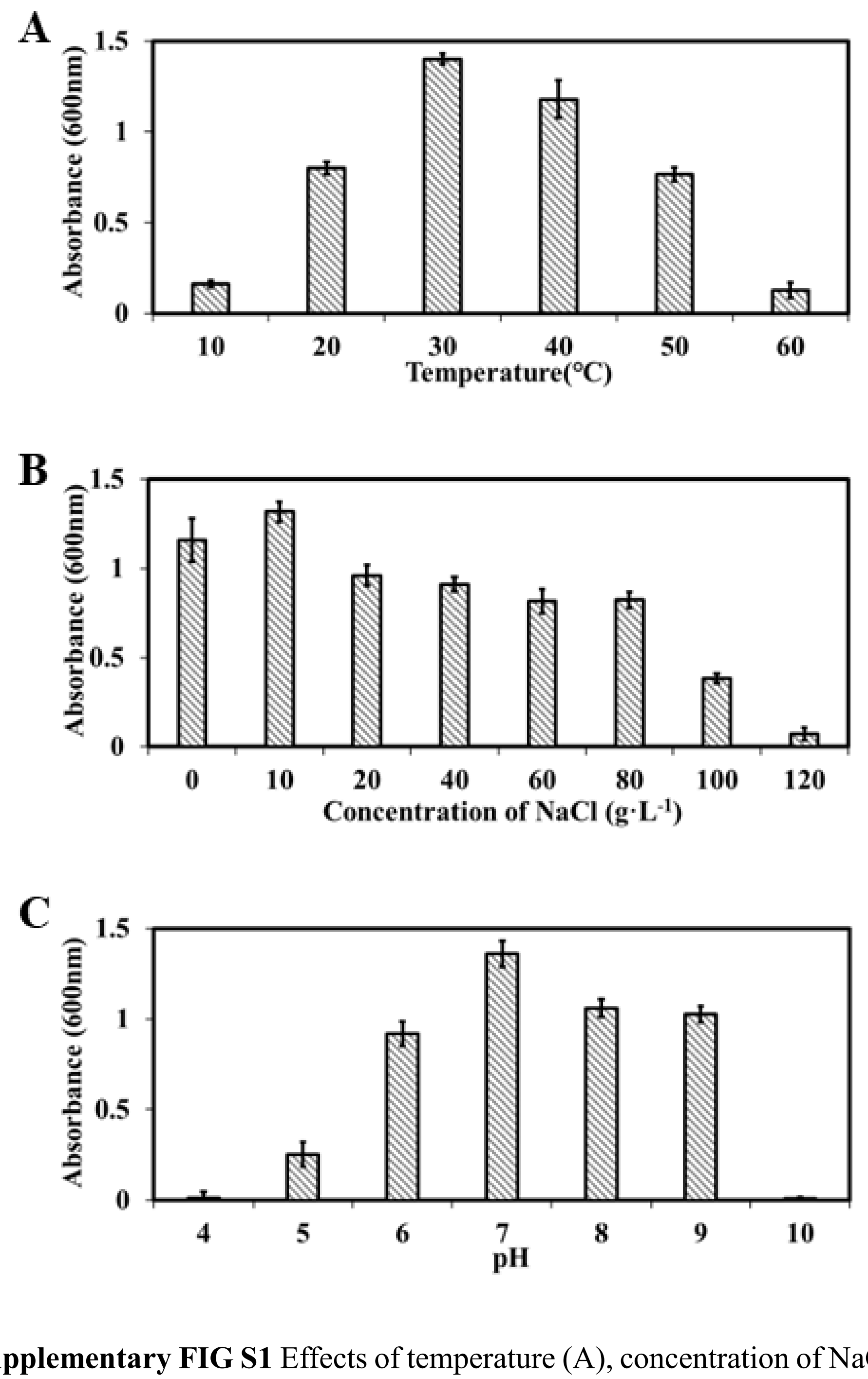

Supplementary FIG S1 Effects of temperature (A), concentration of NaCl (B), and pH (C) on the growth of ZH07.

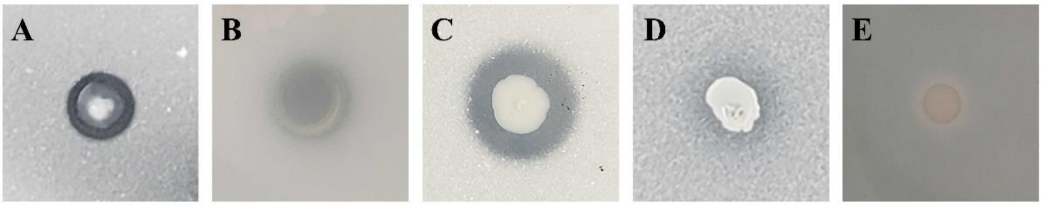

Supplementary FIG S2 Nitrogen fixation activity was tested on N-free Ashby medium (A); mineral phosphate solubilization activity was assayed on inorganic phosphorus medium (B); organic phosphate solubilization potential was assayed on egg yolk medium (C); potassium dissolution activity was assayed on Alexandrov medium (D); siderophore production activity was tested on Chrome azurol S Assay medium (E).

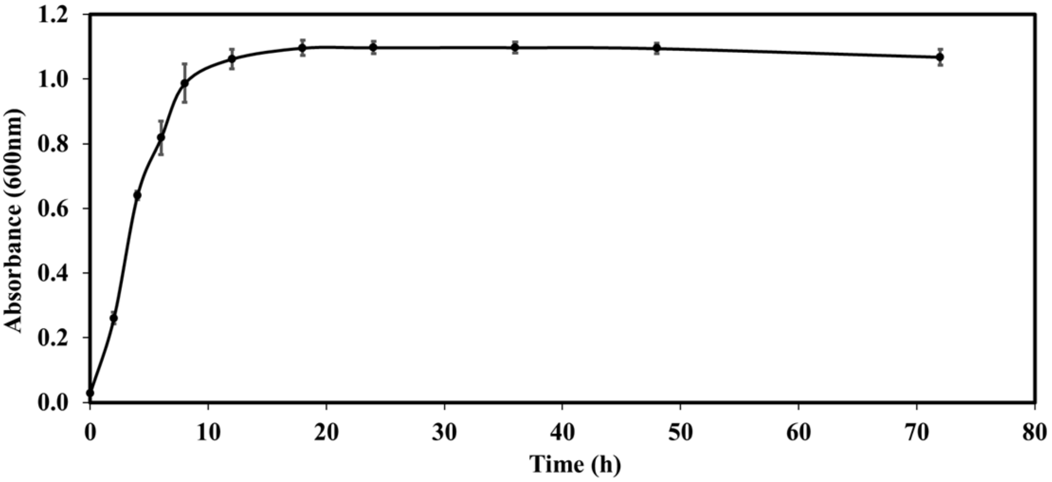

Supplementary FIG S3 Cell growth of strain ZH07 in LB liquid medium.

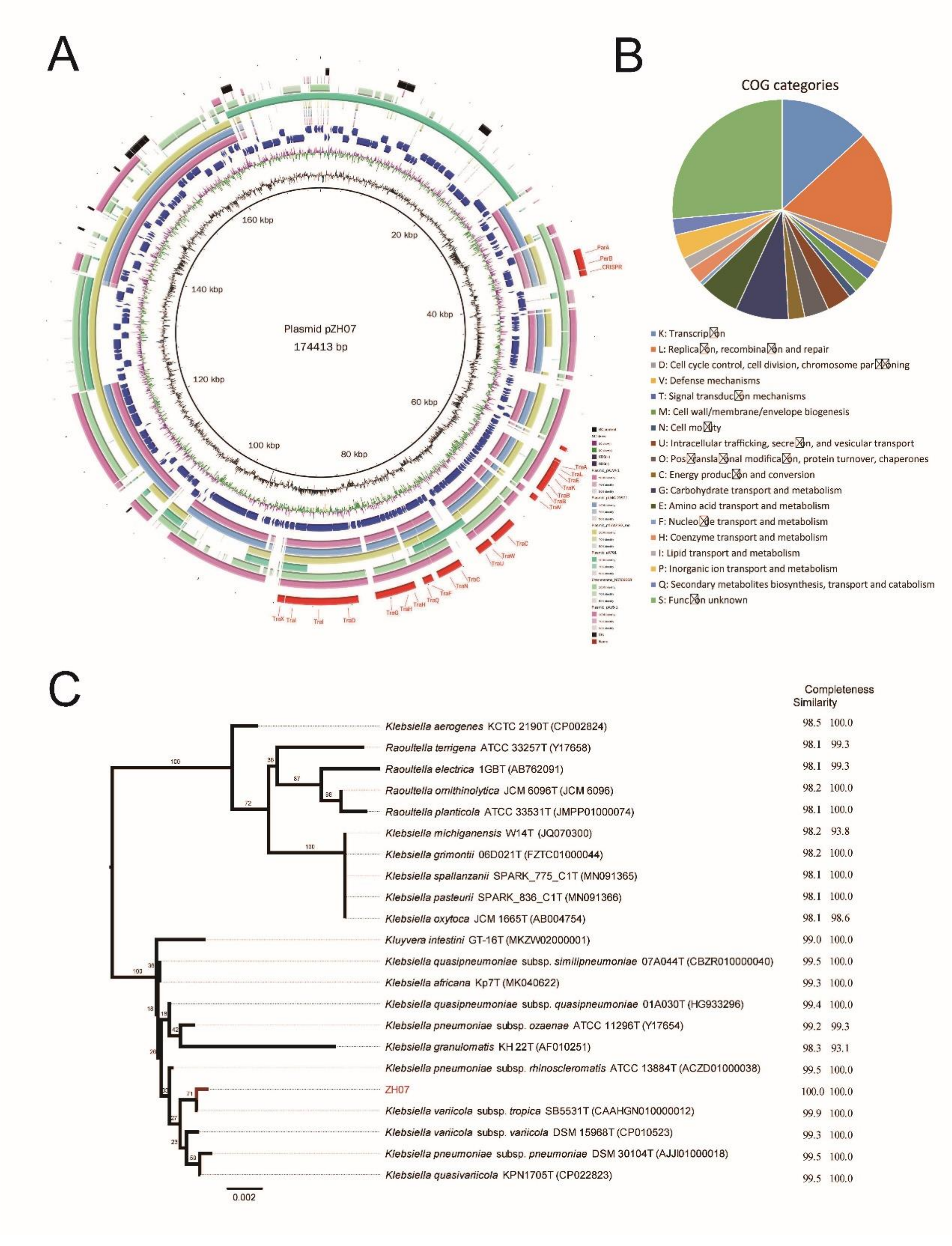

Supplementary FIG S4 Structure and functional analysis of the plasmid pZH07. A. Circular comparison between the plasmid pZH07and other reported similar sequences. A BLASTn search of the NCBI Non-Redundant (NR) database using the full plasmid gene sequences was performed. The six sequences with the largest number of homologs were collected (Table S2). Rings represent the following features labeled from inside

to outside: ring 1, GC content; ring 2, GC-skew; rings 3 and 4, blue arrows correspond to plus-strand CDS and minus-stand CDS; rings 5–10, circular comparison of pKLVA-1, pLMG-23571, p15WZ-82_res, pKP91, NCTC9668, and pKp5-1, respectively; ring 11-12, blocks correspond to ISs and T4SS. B. Distribution of COG categories for plasmid pZH07. C. Phylogenetic tree based on 16S rRNA sequences obtained by the maximum likelihood (ML) method with 1000 replicates.

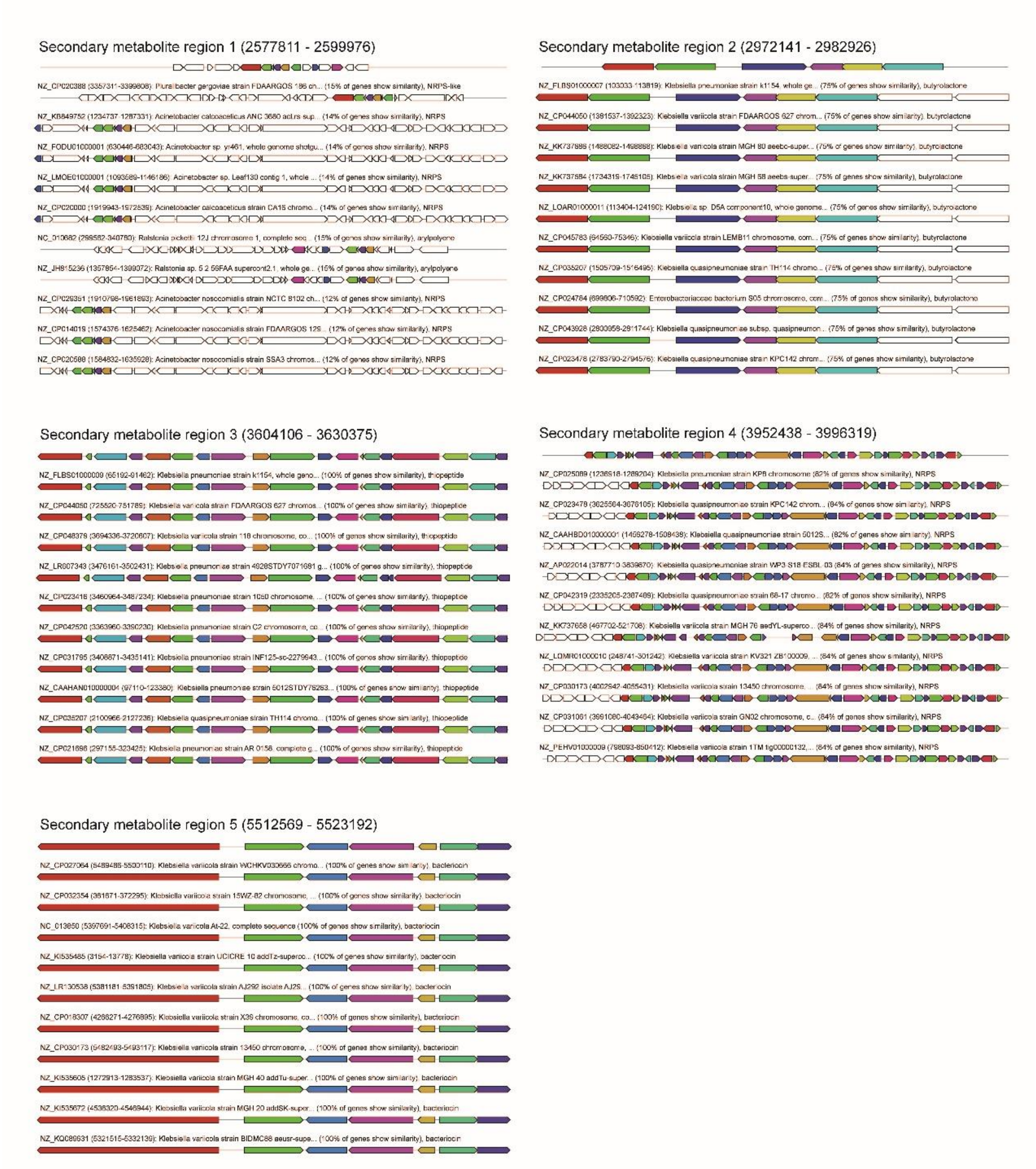

Supplementary FIG S5 Comparative analysis of biosynthetic gene clusters for secondary metabolism from ZH07 and other genomes. Different genes are in different colors, and genes with the same color are homologous to each other.
